# Contributions of linkage disequilibrium and co-segregation information to the accuracy of genomic prediction

**DOI:** 10.1186/s12711-016-0255-4

**Published:** 2016-10-11

**Authors:** Xiaochen Sun, Rohan Fernando, Jack Dekkers

**Affiliations:** Department of Animal Science and Center for Integrated Animal Genomics, Iowa State University, Ames, IA 50011 USA

## Abstract

**Background:**

Traditional genomic prediction models using multiple regression on single nucleotide polymorphisms (SNPs) genotypes exploit associations between genotypes of quantitative trait loci (QTL) and SNPs, which can be created by historical linkage disequilibrium (LD), recent co-segregation (CS) and pedigree relationships. Results from field data analyses show that prediction accuracy is usually much higher for individuals that are close relatives of the training population than for distantly related individuals. A possible reason is that historical LD between QTL and SNPs is weak and, for close relatives, prediction accuracy of SNP models is mainly contributed by pedigree relationships and CS. Information from pedigree relationships decreases fast over generations and only contributes to within-family prediction. Information from CS is affected by family structures and effective population size, and can have a substantial contribution to prediction accuracy when modeled explicitly.

**Results:**

In this study, a method to explicitly model CS was developed by following the transmission of putative QTL alleles using allele origins at SNPs. Bayesian hierarchical models that combine information from LD and CS (LD-CS model) were developed for genomic prediction in pedigree populations. Contributions of LD and CS information to prediction accuracy across families and generations without retraining were investigated in simulated half-sib datasets and deep pedigrees with different recent effective population sizes, respectively. Results from half-sib datasets showed that when historical LD between QTL and SNPs is low, accuracy of the LD model decreased when the training data size is increased by adding independent sire families, but accuracies from the CS and LD-CS models increased and plateaued rapidly. Results from deep pedigree datasets show that the LD model had high accuracy across generations only when historical LD between QTL and SNPs was high. Modeling CS explicitly resulted in higher accuracy than the LD model across generations when the mating design generated many close relatives.

**Conclusions:**

Our results suggest that modeling CS explicitly improves accuracy of genomic prediction when historical LD between QTL and SNPs is low. Modeling both LD and CS explicitly is expected to improve accuracy when recent effective population size is small, or when the training data include many independent families.

**Electronic supplementary material:**

The online version of this article (doi:10.1186/s12711-016-0255-4) contains supplementary material, which is available to authorized users.

## Background

The feasibility of obtaining genotypes of dense single nucleotide polymorphisms (SNPs) with genome-wide coverage has improved accuracy of estimated breeding values by genomic prediction [1–6]. To date, most statistical models for genomic prediction are based on multiple regression of phenotypes on SNP genotype covariates (SNP models). The estimated SNP effects are used to predict genomic estimated breeding values (GEBV) for selection candidates, which are usually progeny of individuals in the training population [7]. Linkage disequilibrium (LD) between quantitative trait loci (QTL) and SNPs was initially thought to be the only source of genetic information that contributes to accuracy of genomic prediction using SNP models, until [8] and [9] showed that co-segregation (CS) of QTL with SNPs and pedigree relationships that are implicitly captured by SNP genotypes also contribute to prediction accuracy.

Co-segregation is an important source of information that contributes to accuracy of genomic prediction [9, 10]. Alleles co-segregate when they originate from the same parental chromosome. Thus, in this study, CS is defined as a non-random association between the grand-parental allele origins of two linked loci. For instance, the maternal alleles of an individual at two loci co-segregate when both alleles originate from the same grand maternal chromosome [11, 12]. With high-density genotyping, the probability that a QTL allele co-segregates with its two adjacent SNP alleles is high. For example, the average distance between adjacent SNPs on the Illumina Bovine SNP50 BeadChip is 50 kb [13, 14], and the average recombination rate between two adjacent SNPs is only around 0.0005 per meiosis, assuming a typical crossover rate of 1 % per million base pair.

In analyses of field datasets using SNP models, high accuracy of genomic prediction has been mainly observed among close relatives [1, 2, 15, 16], and prediction accuracy decreases rapidly when the validation individuals are separated from training individuals by more generations [5, 16–18]. The latter does not agree with results from simulation studies in which the LD between QTL and SNPs was high [7–9, 19]. These results suggest that LD between QTL and SNPs is low in current livestock populations, and that prediction accuracy of the SNP model mainly comes from CS and pedigree relationships that are implicitly captured by SNP genotypes [8, 10, 16, 17, 20].

**Fig. 1:**
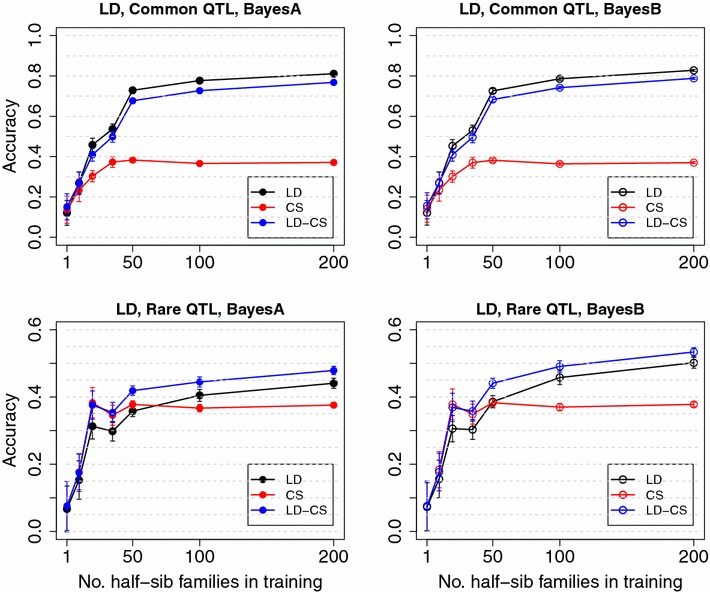
Mean accuracy with different numbers of half-sib families in training with high historical LD. LD, the LD model; CS, the CS model; LD-CS, the LD-CS model. *Top panel*, the Common QTL scenario, *Bottom panel*, the Rare QTL scenario. *Left panel*, BayesA, *right panel*, BayesB

**Fig. 2:**
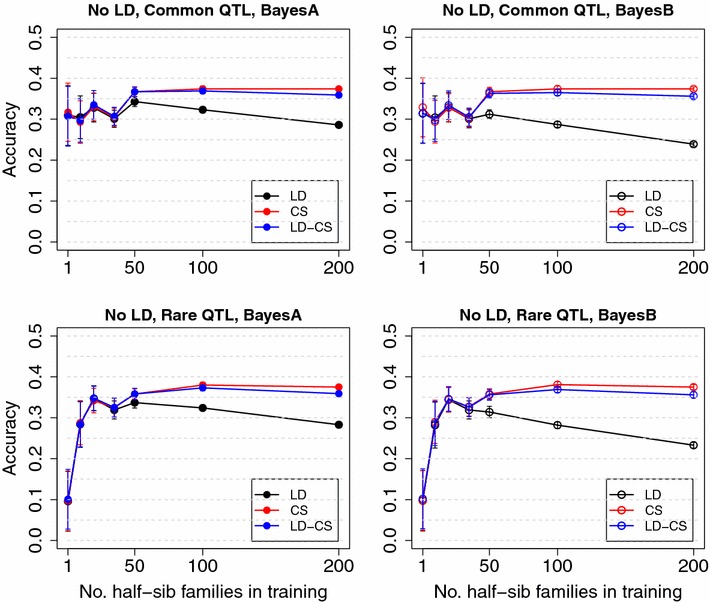
Mean accuracy with different numbers of half-sib families in training with no historical LD. LD, the LD model; CS, the CS model; LD-CS, the LD-CS model. *Top panel*, the Common QTL scenario, *Bottom panel*, the Rare QTL scenario. *Left panel*, BayesA, *right panel*, BayesB

Simulation studies have shown that both LD and CS information contribute to prediction accuracy of the genomic best linear unbiased prediction (GBLUP) model [9]. Information from historical LD was persistent across generations and contributed to prediction accuracy across families and across validation generations. CS information that is captured implicitly by GBLUP was not persistent across families or generations, and its contribution to prediction accuracy decreased when the number of unrelated families increased in the training population [9]. Simulation studies of an aquaculture breeding program [21] showed that the contribution of CS information to prediction accuracy was similar across a wide range of SNP densities, while the contribution of LD information dropped significantly with decreasing SNP density, indicating that the accuracy due to CS was not affected by the level of LD. In a study using data from Italian Brown Swiss bulls, the effect of LD and CS information on prediction accuracy was investigated for the GBLUP model, with the covariance structure of genomic breeding values constructed either using LD or CS information at SNPs [10]. The GBLUP model that fitted both LD and CS had a similar accuracy as that fitting only CS, which was slightly higher than that fitting only LD [10]. Their results also suggest that when historical LD between QTL and SNPs is low, prediction accuracy for closely related individuals mainly comes from CS instead of LD.

Although LD between SNPs has been shown to be sizable in livestock populations [14, 22–24], LD between SNPs and unobservable QTL can be much lower than LD between SNPs, which is probably due to the difference in minor allele frequencies (MAF) of SNPs and QTL. QTL for economically important traits are likely to have low MAF either because the traits have been subject to directional selection for a long time [25–27], or because some QTL are the result of mutations that occurred more recently than the mutations that caused SNPs [25, 27, 28]. SNPs included on SNP chips usually have moderate to high MAF due to ascertainment bias from sequencing and prototype genotyping of reference samples [13]. Since LD between loci that have different MAF is low, LD information contributes little to prediction accuracy when most QTL have much lower MAF than SNPs (Detailed discussion is provided in Additional file 1). Modeling CS explicitly can increase accuracy when historical LD is low because CS information follows transmission of QTL alleles among related individuals, which is independent of the level of LD between QTL and SNPs.

**Fig. 3:**
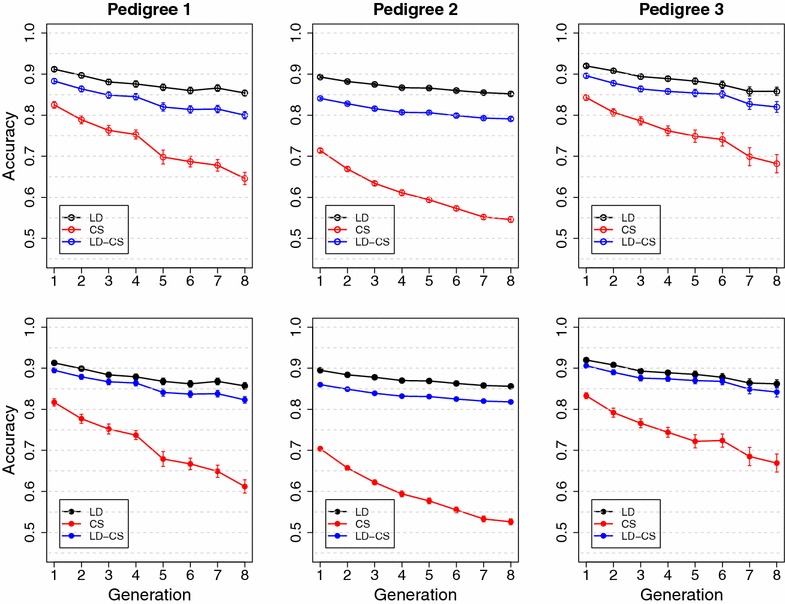
Mean accuracy in three simulated pedigrees in the Common QTL scenario with high historical LD. LD, the LD model; CS, the CS model; LD-CS, the LD-CS model. *Top panel*, BayesA; *bottom panel*, BayesB

**Fig. 4:**
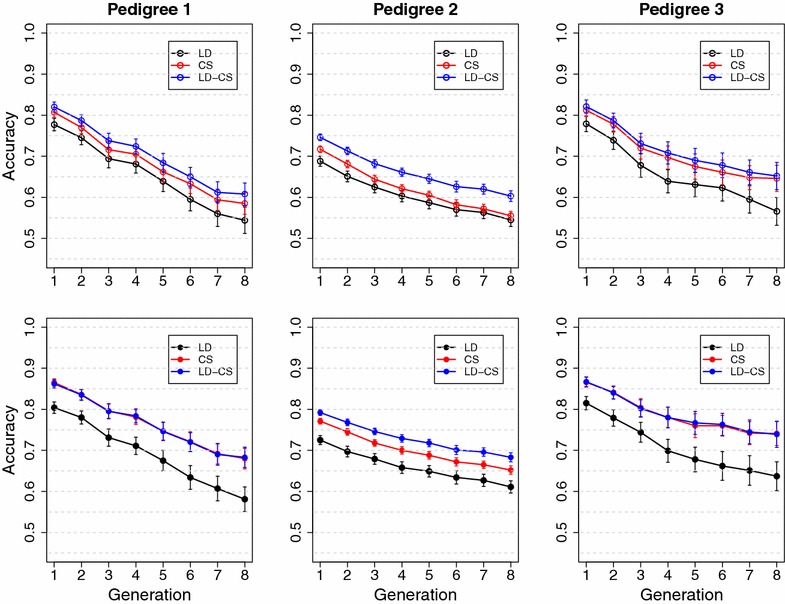
Mean accuracy in three simulated pedigrees in the Rare QTL scenario with high historical LD. LD, the LD model; CS, the CS model; LD-CS, the LD-CS model. *Top panel*, BayesA; *bottom panel*, BayesB

Explicit modeling of CS information for genomic prediction was proposed and developed by Luan et al. [10]. In Luan et al. [10], CS was modeled at each SNP locus using the method in Fernando and Grossman [29], and a realized relationship matrix using CS information was constructed by averaging across all SNPs with equal weights. The method for modeling CS in Luan et al. [10] can be improved in two aspects. First, since CS signals span long genomic distances, modeling CS across multiple SNPs is expected to capture the same amount of CS information as modeling at each SNP, but modeling across multiple loci can substantially improve computational efficiency. Second, Luan et al. [10] assumed that the contribution of CS information at each SNP was the same. The variance at QTL can vary due to differences in allele frequencies and effects at the QTL, and these variances can be treated as unknowns and marginalized in a Bayesian analysis. In this study, a new method is developed to model CS explicitly. The CS model follows the transmission of putative QTL within 1-cM genomic windows. A detailed description of a Bayesian hierarchical model for genomic prediction using CS is provided, and a Gibbs sampling algorithm for prediction of breeding values is derived.

Persistence of prediction accuracy across validation generations without retraining (long-term accuracy) is an important criterion to evaluate contributions of LD and CS information to prediction accuracy. Habier et al. [9] showed that LD information was more persistent than the CS information that is implicitly captured by SNP genotypes because CS information decays across generations due to recombination within large chromosome segments. Modeling CS explicitly at small putative QTL regions is expected to improve accuracy across generations because recombination is less likely to happen within small chromosome segments. The contribution of CS information to accuracy across generations by modeling CS explicitly has not been studied.

Recent effective population size ($N_e$) is another important factor that affects the contribution of CS information to long-term accuracy. For a given size of the training and validation populations, individuals are more closely related when recent $N_e$ is smaller and CS information is expected to contribute more to long-term accuracy in that case for two reasons. First, CS is generated as associations between loci over long chromosome regions. Smaller recent $N_e$ causes higher CS due to stronger drift and selection of alleles in recent generations. Second, with smaller recent $N_e$, fewer founder alleles are each inherited by relatively more offspring, and, thus, the values of founder alleles can be estimated more accurately when more data is available for each allele. In livestock populations, recent $N_e$ is affected by the mating design and can vary greatly between breeding programs. Therefore, it is important to study the effect of recent $N_e$ on the contribution of CS information to long-term accuracy of genomic prediction.

The objectives of this study were (1) to develop a Bayesian statistical method to model CS explicitly by following the transmission of putative QTL alleles of pedigree founders, (2) to investigate contributions of LD and CS information to the accuracy of genomic prediction across unrelated families and validation generations without re-training, and (3) to investigate the effects of historical LD, recent $N_e$, and MAF of QTL on the advantage of modeling LD and CS explicitly to improve prediction accuracy.

**Fig. 5:**
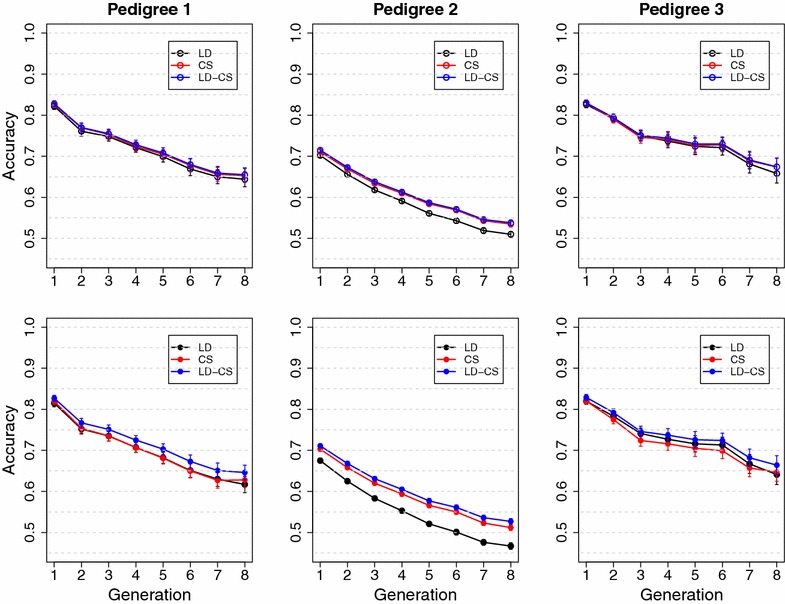
Mean accuracy in three simulated pedigrees in the Common QTL scenario with no historical LD. LD, the LD model; CS, the CS model; LD-CS, the LD-CS model. *Top panel*, BayesA; *bottom panel*, BayesB

**Fig. 6:**
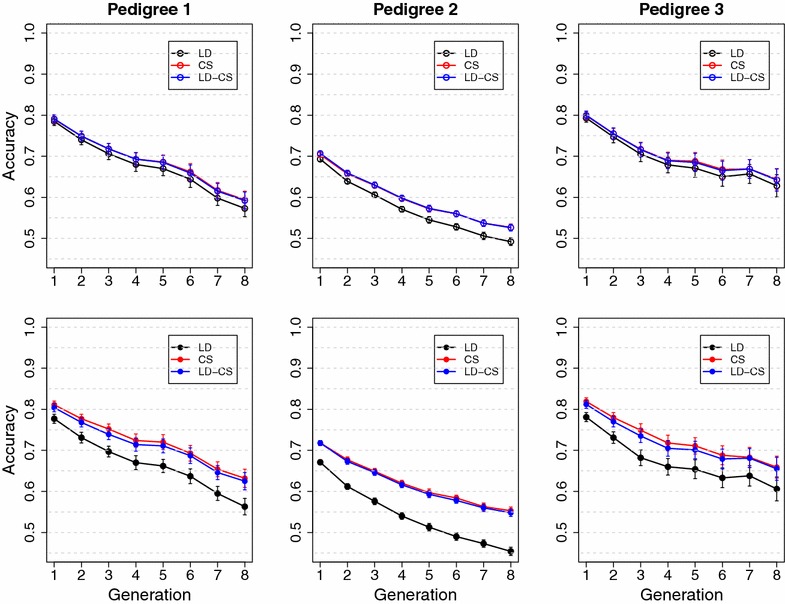
Mean accuracy in three simulated pedigrees in the Rare QTL scenario with no historical LD. LD, the LD model; CS, the CS model; LD-CS, the LD-CS model. *Top panel*, BayesA; *bottom panel*, BayesB

## Methods

### Genomic prediction models using LD, CS and combined LD-CS information

Definitions of LD, CS, and pedigree relationship are presented for a population with known pedigree information following Habier et al. [12]. LD is defined as all non-random associations between allele states in pedigree founders. These associations result in greater similarity between individuals that have the same marker allele states due to QTL that are in LD with the markers. CS is defined as non-random association between allele origins in pedigree generations. These associations result in higher covariances between relatives that have the same marker allele origins than those conditional only on pedigree relationships due to CS between QTL and markers. Similarly, relatives that have different marker allele origins will have lower covariances than those conditional only on pedigree relationships. Once LD is defined within pedigree founders, all the “new” associations created in pedigree generations can be explained by co-segregation and pedigree relationships. Since pedigree relationships quickly dissipate with generations, their contribution to long-term accuracy is small [8, 12]. Therefore, this study only focuses on the contributions of LD and CS information.

Following Meuwissen et al. [7], the statistical model that uses LD information for prediction of GEBV for a quantitative trait is written as:

1 $$\begin{aligned} \mathbf {y} = \mathbf {X}\varvec{{\upbeta }} + \mathbf {Z}\varvec{{\upalpha }} + \mathbf {e}, \end{aligned}$$

where $\mathbf {y}$ is an $n\times 1$ vector of trait phenotypes of *n* training individuals, $\varvec{{\upbeta }}$ is a vector of non-genetic fixed effects, $\mathbf {X}$ is the design matrix for fixed effects, $\mathbf {Z}$ is an $n\times m$ matrix with each row containing genotypes (coded as 0/1/2) at *m* SNPs of each training individual, $\varvec{{\upalpha }}$ is an $m\times 1$ vector of allele substitution effects of the *m* SNPs, and $\mathbf {e}$ is an $n\times 1$ vector of residuals. Informative prior distributions are usually given to $\varvec{{\upalpha }}$ to allow simultaneous estimation of all SNP effects.

In the LD model (1), QTL effects are not explicitly fitted but SNPs are used as surrogates for QTL due to LD. The genotypic value at the QTL that is captured by surrounding SNP genotypes can be viewed as the conditional expectation of this genotypic value given SNP genotypes. When LD between QTL and SNPs is not complete, the true genotypic value at the QTL deviates from its conditional expectation. Therefore, under low LD, the LD model can only capture part of the genetic variance at QTL.

The CS model is given by

2 $$\begin{aligned} \mathbf {y} = \mathbf {X}\varvec{{\upbeta }}+\sum _{j=1}^{n_q}\mathbf {W}_j\mathbf {v}_j+\mathbf {e}, \end{aligned}$$

where $\mathbf {y}$ is an $n\times 1$ vector of trait phenotypic values of *n* training individuals, $\varvec{{\upbeta }}$ and $\mathbf {X}$ are the same as in the LD model (1), $\mathbf {v}_j$ is a vector of the values of founder alleles at the *j*th putative QTL, with $n_q$ the number of putative QTL, $\mathbf {W}_j$ is the covariate matrix for $\mathbf {v}_j$, and $\mathbf {e}$ is an $n\times 1$ vector of residuals. As in the LD model, informative prior distributions are given to $\mathbf {v}_j$’s to allow simultaneous estimation of the value of founder alleles. Definitions of the value of founder alleles $\mathbf {v}_j$ and their covariates $\mathbf {W}_j$ are given in the next section.

The model that fits both LD and CS (LD-CS model) includes the LD and CS terms from models (1) and (2),

3 $$\begin{aligned} \mathbf {y} = \mathbf {X}\varvec{{\upbeta }}+\mathbf {Z}\varvec{{\upalpha }}+\sum _{j=1}^{n_q}\mathbf {W}_j\mathbf {v}_j+\mathbf {e}. \end{aligned}$$

In the LD-CS model (3), the conditional expectation of the genotypic value at a putative QTL is captured by surrounding SNP genotypes in the LD term, while the genotypic value at a putative QTL are explicitly fitted in the CS term. When LD between QTL and SNPs is not complete, deviations between QTL genotypic values and their conditional expectations on SNP genotypes are captured by the CS term. Therefore the LD-CS model (3) is expected to capture most genetic variance at QTL under incomplete LD.

**Fig. 7:**
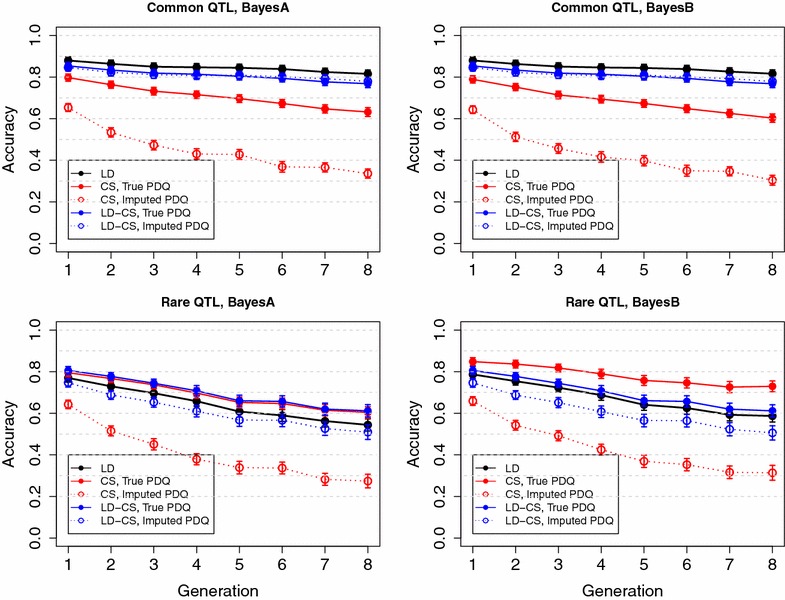
Mean accuracy in simulated pedigree 1 with high historical LD using true or imputed allele origins. LD, the LD model; CS, the CS model; LD-CS, the LD-CS model. *Top panel*, Common QTL scenario; *bottom panel*, Rare QTL scenario

**Fig. 8:**
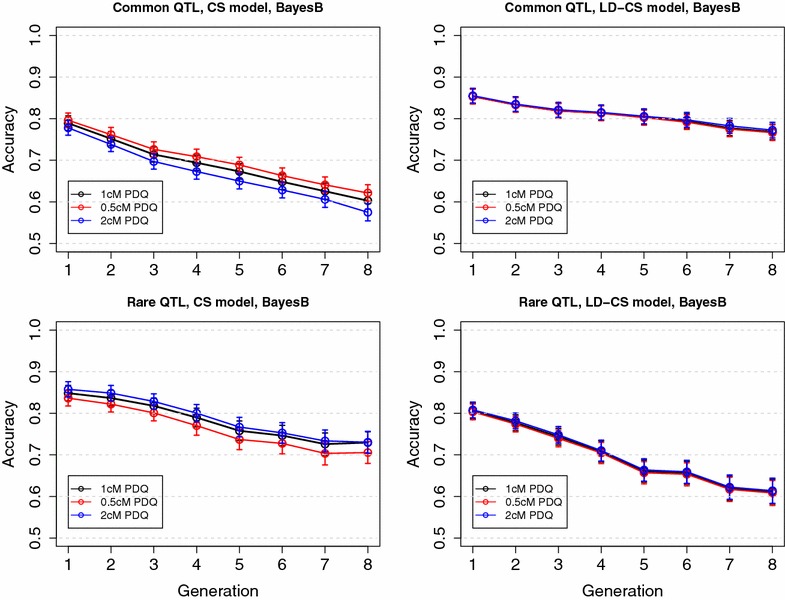
Mean accuracy in simulated pedigree 1 with high historical LD when fitting putative QTL at genome windows with different lengths. LD, the LD model; CS, the CS model; LD-CS, the LD-CS model. *Top panel*, Common QTL scenario; *bottom panel*, Rare QTL scenario. Results were from BayesB

### Statistical modeling of CS information

Co-segregation of alleles at two loci means that these alleles share identical grand-parental allele origins, i.e. they both originate from the same chromosome of a parent. The indicator of parental allele origin at one locus is a Bernoulli variable. In this study, the allele origin indicator equals 0 if it originates from its grand-maternal allele, and 1 if it originates from its grand-paternal allele. When allele origins of parents and offspring at a SNP are known, the probability that the allele origin of a putative QTL linked to the SNP is grand-paternal (equals 1) can be calculated using recombination rates between QTL and SNPs, which is termed the probability of descent of the QTL allele (PDQ). Suppose that allele origins are known for an individual’s maternal alleles at two SNPs $\text {M}_1$ and $\text {M}_2$. Then, assuming no interference, the PDQ at the putative QTL is calculated as follows when the origins of both SNP alleles are the mother’s maternal allele, i.e. $O^m_1=0$ and $O^m_2=0$,

4 $$\begin{aligned} \text {Pr}(O^m_{\text {Q}}=0|O^m_1=0,O^m_2=0)=\frac{\text {Pr}(O^m_1=0,O^m_{\text {Q}}=0,O^m_2=0)}{\text {Pr}(O^m_1=0,O^m_2=0)}=\frac{(1-r_1)(1-r_2)}{1-r_{12}}, \end{aligned}$$

where $O^m_i$ is the maternal allele origin at $\hbox {M}_i$ for $i=1,2$, $O^m_\text {Q}$ is the maternal allele origin at the QTL, $r_1$ is the recombination rate between $\hbox {M}_1$ and QTL, $r_2$ is the recombination rate between QTL and $\hbox {M}_2$, and $r_{12}$ is the recombination rate between $\hbox {M}_1$ and $\hbox {M}_2$. Recombination rates $r_1$, $r_2$ and $r_{12}$ can be calculated from the map distance between $\hbox {M}_1$ and $\hbox {M}_2$ using mapping functions [30].

In most cases, positions of QTL are not known, and genome-wide CS information is modeled for putative QTL within each non-overlapping genomic window of a certain length. In this study, putative QTL were fitted at genome windows of lengths 0.5, 1 and 2 cM, respectively, to study the impact of window length on the accuracy of the CS and LD-CS models. Within each genomic window, the putative QTL is assumed to be located at the midpoint of SNPs $\hbox {M}_1$ and $\hbox {M}_2$ that are flanking the window. It follows that $r_1=r_2=0.5[1-\exp (-d_{\text {M}_1\text {M}_2})]$ and $r_{12}=0.5[1-\exp (-2d_{\text {M}_1\text {M}_2})]$, where $d_{\text {M}_1\text {M}_2}$ is the distance between $\hbox {M}_1$ and $\hbox {M}_2$ in Morgan. Transmission of putative QTL alleles is followed by PDQ calculated using allele origins of $\hbox {M}_1$ and $\hbox {M}_2$. When there is no recombination between $\hbox {M}_1$ and $\hbox {M}_2$, the PDQ of the putative QTL is either 0 or 1, indicating the transmission of grand maternal or paternal QTL allele, respectively. When recombination occurs between $\hbox {M}_1$ and $\hbox {M}_2$, the PDQ of the putative QTL is 0.5, meaning that the value of the recombinant QTL allele is the average of the values of the two parental QTL alleles.

The method for modeling CS uses PDQ to follow the transmission of founder alleles at putative QTL. The true breeding value (TBV) of an individual is the summation of the value of its maternal and paternal alleles (denoted as $v^m$ and $v^p$, respectively) of putative QTL across the genome. At each putative QTL, the values of founder QTL alleles were assumed independent. The values of non-founder QTL alleles are linear combinations of the values of founder QTL alleles, with covariates determined by the PDQ. The covariates of maternal and paternal QTL alleles of a non-founder individual *i* (${\mathbf {w}'}_i^m$ and ${\mathbf {w}'}_i^p$, respectively) were calculated recursively as

5 $$\begin{aligned} {\mathbf {w}'}_i^m = \text {PDQ}^m_i{\mathbf {w}'}_{\text {dam}}^m + (1-\text {PDQ}^m_i){\mathbf {w}'}_{\text {dam}}^p, \end{aligned}$$

and

6 $$\begin{aligned} {\mathbf {w}'}_i^p = \text {PDQ}^p_i{\mathbf {w}'}_{\text {sire}}^m + (1-\text {PDQ}^p_i){\mathbf {w}'}_{\text {sire}}^p, \end{aligned}$$

where $\text {PDQ}^m_i$ ($\text {PDQ}^p_i$) is the maternal (paternal) PDQ for non-founder *i*, and ${\mathbf {w}'}_{\text {dam}}^m$ (${\mathbf {w}'}_{\text {sire}}^p$) is the dam’s maternal (sire’s paternal) covariates for founder QTL alleles. Vectors ${\mathbf {w}'}_{\text {dam}}^m$ and ${\mathbf {w}'}_{\text {sire}}^p$ at each QTL have dimension equal to twice the number of pedigree founders.

Vectors ${\mathbf {w}'}_i^m$ and ${\mathbf {w}'}_i^p$ comprise the rows in the incidence matrix $\mathbf {W}_{\text {H}}$ that relates the values of QTL alleles of all individuals with those of founders. The incidence matrix that relates TBV with the values of founder QTL alleles ($\mathbf {W}$) was derived by the summation of every two rows in $\mathbf {W}_{\text {H}}$ that correspond to the paternal and maternal QTL alleles of the same individual, i.e.

$$\begin{aligned} \mathbf {W} = \big (\mathbf {I}_n\otimes [1,1]\big )\times \mathbf {W}_\text {H}, \end{aligned}$$

where $\mathbf {I}_n$ is the identity matrix with dimension *n*. The vector of TBV of all individuals ($\mathbf {g}_{\text {CS}}$) can be written as

$$\begin{aligned} \mathbf {g}_{\text {CS}} = \mathbf {W}\mathbf {v}, \end{aligned}$$

where $\mathbf {v}$ is the vector of values of founder alleles at all putative QTL.

**Table 1:** Minor allele frequencies (MAF) of QTL and SNPs and the level of historical linkage disequilibrium (LD) in the base population of simulated scenarios

Scenario	Common QTL$^{\mathrm{a}}$		Rare QTL$^{\mathrm{b}}$	
	High LD$^{\mathrm{c}}$	No LD$^{\mathrm{d}}$	High LD	No LD
MAF of QTL	0.01–0.5		0.01–0.06	
MAF of SNPs	0.06–0.5			
LD between QTL and SNPs	>0	=0	≈0	=0
LD between SNPs	>0	=0	>0	=0

$^{\mathrm{a}}$ The scenario with MAF of QTL between 0.01 and 0.50, and MAF of SNPs between 0.06 and 0.50

$^{\mathrm{b}}$ The scenario with MAF of QTL between 0.01 and 0.06, and MAF of SNPs between 0.06 and 0.50

$^{\mathrm{c}}$ The scenario with high LD in the base population created by historical generations

$^{\mathrm{d}}$ The scenario with linkage equilibrium in the base population by independently sampling genotypes of QTL and SNPs

### Prediction of breeding values

Method BayesA and BayesB were used to estimate SNP allele substitution effects and values of founder QTL alleles. Details of Bayesian inference using BayesA and BayesB are given in Additional file 2. The value of $\pi _{\text {SNP}}$ for BayesB in the LD and LD-CS models was calculated as $1-\frac{\text {Number of QTL}}{\text {Number of SNPs}}$. The value of $\pi _{\text {CSE}}$ for BayesB in the CS and LD-CS models was 0.95, indicating the proportion of founder alleles that have an ignorable effect on TBV. The Gibbs sampler was run for 21,000 iterations, with the first 1000 discarded as burn-in. Point estimates of SNP effects and values of founder alleles at putative QTL were posterior means calculated from the MCMC samples.

### Simulation of the base population

Contributions of LD and CS information to prediction accuracy were investigated using simulated datasets of paternal half-sib designs with different numbers of independent sire families, and extended pedigrees with three mating designs that differed in recent $N_e$. Parents of half-sibs and founders of extended pedigrees were random samples from the same base population, following closely the simulations of Habier et al. [8, 16] and Sun et al. [31].

The simulated genome comprised two chromosomes, each 1 Morgan long. Each chromosome was evenly covered by 4000 SNPs. Fifty candidate QTL were randomly positioned within each cM of the genome. The mutation rate for QTL and SNPs was $2.5\times 10^{-5}$ per meiosis per locus. The number of crossovers per chromosome was sampled from a Poisson distribution with mean 1.0, and the positions of the crossovers were sampled from a uniform distribution.

Two scenarios were simulated for historical LD between SNPs in the base population. In the scenario of high historical LD between SNPs, the base population was generated as follows. The initial generations comprised a population with $N_e = 500$ that was randomly mated for 500 generations to generate LD between closely linked loci, after which the population was shrunk to $N_e = 200$ and randomly mated for another 100 generations to create LD over longer genetic distances. In the next 10 generations, the population was linearly scaled up to an actual size of 2000 as the base population. In the scenario with no historical LD between SNPs, a population of actual size 2000 was generated as base population with SNP and QTL alleles randomly sampled with frequency 0.5. This resulted in a population that was both in linkage equilibrium and in Hardy-Weinberg equilibrium.

In the base population, 2000 SNPs with MAF higher than 0.05 on each chromosome and one segregating QTL within each cM of the genome were sampled according to their MAF, depending on the scenario (Table 1). In the Common QTL scenario, all QTL had MAF between 0.01 and 0.5. In the Rare QTL scenario, all QTL had MAF between 0.01 and 0.06. Additive QTL effects were randomly sampled from a standard normal distribution. The TBV were obtained as the summation of all QTL allele values for a given individual. Allele substitution effects of QTL were scaled in the base population to achieve a genetic variance equal to 4.29. Normally distributed random errors with mean 0 and variance 10.0 were added to TBV to generate phenotypes for a quantitative trait with a narrow sense heritability of 0.3.

For each pedigree, 50 replicated datasets were independently simulated for each scenario in Table 1. All replicated datasets used the same initial SNP positions but had different randomly sampled QTL effects and, after selection of loci based on MAF, had different positions of QTL and SNPs.

### Simulation of half-sib datasets

To study contributions of LD and CS information to prediction accuracy across unrelated families, paternal half-sib families from different numbers of sires were simulated. From the base population, *s* sires and $20\times s$ dams were randomly sampled without replacement as the parents of half-sib offspring. Each of the *s* sires was mated with 20 dams, with each dam producing one offspring. Within each sire family, 10 random half-sib offspring were used in the training population and the other 10 for validation. Independent datasets were generated for different numbers of sire families, $s=1,2,5,10,50,100$ and 200, corresponding to training population sizes of 10, 20, 50, 100, 500, 1000 and 2000, respectively.

### Simulation of pedigree population designs

To study the effect of recent $N_e$ on long-term accuracy due to LD or CS information, three mating designs with different recent $N_e$ were simulated. The mating designs were represented by three pedigrees with 13 non-overlapping generations but different numbers of parents and offspring per mating. The founders (first generation) of all three pedigrees comprised five sires, each mated with 10 dams. Sires and dams of the first generation were randomly sampled from a base population of size 2000. Every mating in the first generation produced six male and six female progeny (second generation).

In pedigree 1, five sires and 50 dams were randomly selected in each generation starting from the 2nd generation. Each sire was mated with 10 dams, each producing six male and six female progeny. Pedigree 1 represents a balanced nested design where a small number of sires was selected in each generation and each sire on average contributed equally to the next generation. The $N_e$ for pedigree 1 was calculated as $N_e = \frac{4\times 5\times 50}{5+50} = 18.2$ [32].

In pedigree 2, all 300 sires and 300 dams from generation 2 were used as parents. Each sire was mated with one dam, producing one male and one female progeny. Pedigree 2 represents an outbred population where all individuals survived, but each individual had a relatively limited contribution to future generations. Since each individual contributes an equal number of gametes to the next generation, and the variance of family sizes is zero, the $N_e$ of pedigree 2 is approximated by $2N = 1200$, where *N* is the actual population size of each generation (600 for pedigree 2) [32].

In pedigree 3, five sires and 70 dams were randomly selected in each generation starting from the second generation. One sire was mated with 50 dams, each dam producing five male and five female progeny, representing an influential sire family. Each of the other four sires was mated with five dams, each dam producing two male and three female progeny, representing four small sire families. Pedigree 3 represents an unbalanced nested design, where the genetics of one individual dominates future generations. The $N_e$ of pedigree 3 was much less than 18.2.

The first five pedigree generations, with size 2455 (pedigree 1 and 2) and 2475 (pedigree 3), were used for training. Each of the following eight generations, with size 600, were used for validation. Prediction accuracy was calculated as the correlation between GEBV and TBV in each validation generation.

In all simulated datasets, allele origins were assumed known without error at all SNPs. Putative QTL were fitted within each 1-cM genome and PDQ were calculated using the simulated true allele origins. In addition, for pedigree 1, allele origins at all SNPs were either assumed known without error, or imputed using the LDMIP software, with default parameter settings [33]. Putative QTL were fitted within every 0.5, 1 and 2 cM and PDQ were calculated using either true or imputed allele origins, to investigate the impacts of length of genomic windows to model CS and unknown allele origins.

## Results

### Half-sib designs

In the Common QTL scenario with high historical LD, prediction accuracy of the LD model increased from less than 0.2 with one half-sib family and quickly plateaued around 0.8 when the number of half-sib families exceeded 50, which corresponds to a training size of 500 (Fig. 1). Accuracy of the LD-CS model could not be distinguished from that of the LD model at all training sizes. Accuracy of the CS model increased from 0.2 and plateaued around 0.4 when the training size exceeded 500, which was much lower than accuracies of the LD or LD-CS model (Fig. 1). These results suggest that when LD between QTL and SNPs is high, the LD model has high accuracy by capturing information from both LD and CS, and modeling CS explicitly in addition to LD does not improve prediction accuracy.

In the Rare QTL scenario with high historical LD between SNPs, the actual level of historical LD between QTL and SNPs was low due to all QTL having much lower MAF than SNPs. Accuracy of the LD model increased with training size from 0.1 to about 0.45, which was much lower than accuracy with the Common QTL scenario (Fig. 1). Accuracy of the CS model also increased with training size and plateaued around 0.4, which was similar to the Common QTL scenario. Accuracy of the LD-CS model increased and became significantly higher than accuracy of both the LD and CS models when the training size exceeded 100 (10 half-sib families) (Fig. 1). These results suggest that when LD between QTL and SNPs is low, the contribution of CS information is more important than when LD between QTL and SNPs is high, and that modeling CS explicitly in addition to LD improves prediction accuracy across unrelated families.

In the Common QTL and Rare QTL scenarios without historical LD, the LD, CS and LD-CS models had similar accuracies when the training size was less than 500 (Fig. 2). The CS and LD-CS models had higher accuracy than the LD model when the training size exceeded 500. Accuracy from the LD model decreased from 0.35 to 0.25 when the training size exceeded 500 (Fig. 2). These results suggest that when there is no historical LD between QTL and SNPs, accuracy of the LD model comes from implicitly capturing CS information, but the ability to capture CS information decreases when a large number of unrelated families are included in the training population. Without historical LD, the CS model has much higher accuracy than the LD model due to explicitly capturing CS information.

### Pedigree mating designs

In the Common QTL scenario with high historical LD, the LD model had higher accuracy than either the CS or LD-CS model (Fig. 3). For all three pedigrees, the LD-CS model had slightly lower accuracy than the LD model, but the CS model had much lower accuracy than the LD model. Accuracies from the LD and LD-CS models only decreased marginally across the eight validation generations, but the accuracy of the CS model decreased rapidly (Fig. 3). These results suggest that when historical LD between QTL and SNPs is high, the LD model has persistently high accuracy across validation generations without retraining by accurately capturing QTL effects. The CS model estimates only the values of founder alleles, and the values of recombinant alleles that are generated across generations cannot be accurately estimated. Accuracies of the LD and LD-CS models were similar for all three pedigrees, because accuracy was mostly contributed by LD that was generated historically, which was not eroded within a limited number of generations.

Reductions in accuracy for the CS model across validation generations were less severe in pedigrees 1 and 3 compared with pedigree 2 (Fig. 3). The reason is that the number of founder alleles was much smaller in pedigrees 1 and 3 than in pedigree 2 and, thus, the values of founder alleles could be estimated more accurately due to more data available per founder allele. Similar trends in prediction accuracy were observed for BayesB compared with BayesA, except that the difference in accuracy between the LD and LD-CS models was smaller for BayesB than for BayesA, especially in pedigrees 1 and 3 (Fig. 3).

In the Rare QTL scenario with high historical LD, the CS and LD-CS models had higher accuracy than the LD model (Fig. 4). The reduction in accuracy across validation generations was larger for the LD model than for the CS and LD-CS models, especially for pedigrees 1 and 3. These results suggest that when LD between QTL and SNPs is low, the accuracy of the LD model mostly comes from capturing CS information, and this information decreases across validation generations due to recombination. In pedigrees 1 and 3, the LD-CS model had slightly higher accuracy than the CS model when using BayesA, but accuracies of the CS and LD-CS models were almost the same when using BayesB (Fig. 4). In pedigree 2, the LD-CS model had significantly higher accuracy than the CS model for both BayesA and BayesB. This is because when recent $N_e$ is large, as in pedigree 2, the CS model has a disadvantage due to a large number of segregating alleles, each with relatively little data that contribute to the estimation of its value. Modeling LD in addition to CS improves prediction accuracy by implicitly capturing extra CS information. In conclusion, the contribution of CS information to prediction accuracy is more important in pedigrees with few parents than in pedigrees with many parents, because with few parents, the value of founder QTL alleles can be estimated more accurately due to more data being available.

In the Rare QTL scenario with high historical LD, accuracy of BayesB was higher than that of BayesA for the CS model (Fig. 4). This is because when QTL alleles have low MAF, the proportion of founder alleles that carry the favorable QTL allele is low. BayesB is more effective than BayesA to accurately estimate the value of the small proportions of founder alleles that carry favorable QTL alleles.

In the Common QTL scenario without historical LD, the LD, CS and LD-CS models had almost the same accuracy in either pedigree 1 or 3; while in pedigree 2, the LD model had much lower accuracy than the CS and LD-CS models (Fig. 5). When there is no historical LD, only CS information contributes to prediction accuracy. Recent LD between linked QTL and SNPs was created quickly within several generations in pedigrees 1 and 3 due to high genetic drift, in which case the LD model can capture as much CS information as the CS model. The creation of recent LD was slower in pedigree 2 due to much less drift compared with pedigrees 1 and 3, and hence the LD model could only capture part of the CS information.

In the Rare QTL scenario without historical LD, the CS and LD-CS models had similar accuracies, which was higher than the accuracy of the LD model (Fig. 6). This is because high LD cannot be created within several recent generations due to the difference in MAF between QTL and SNPs. Method BayesB had higher accuracy than BayesA for the CS and LD-CS models, because the value of the small proportion of founder alleles that carry favorable QTL alleles was estimated more accurately using BayesB than BayesA. In contrast, method BayesB had lower accuracy than BayesA for the LD model, because more SNPs were fitted by BayesA than by BayesB and hence captured more CS information [9].

### Impact of unknown allele origins

To study the impact of unknown allele origins on the accuracy of the CS and LD-CS models, prediction accuracy was also compared for models using simulated true allele origins and allele origins imputed by the LDMIP software [33] in the simulated datasets of Pedigree 1 with high historical LD. In the Common QTL scenario, the CS model using imputed allele origins had lower accuracy when using imputed allele origins than when using true allele origins, and the accuracy decreased faster over generations, while the LD-CS model had similar accuracy using either imputed or true allele origins (Fig. 7). The reason is that, when there was high historical LD, the accuracy was mostly contributed by LD, which was hardly affected by the accuracy of allele origins. In the Rare QTL scenario, the CS and LD-CS models had lower accuracy when using imputed allele origins than when using true allele origins (Fig. 7). The reason was that, when there was no historical LD, the accuracy was mostly contributed by CS, which was significantly affected by the accuracy of allele origins.

### Impact of the length of the genomic window used to model CS

To study the impact of the length of the genomic window used to model CS on the accuracy of the CS and LD-CS models, values of founder alleles at putative QTL were fitted at genome windows of lengths 0.5, 1 and 2 cM, respectively, in the simulated datasets of pedigree 1 with high historical LD. Prediction accuracies of CS models that used different window lengths was similar, although shorter window lengths tended to have slightly higher accuracy in the Common QTL scenario but lower accuracy in the Rare QTL scenario (Fig. 8). Prediction accuracies of LD-CS models that used different window lengths was almost identical in the Common and Rare QTL scenarios (Fig. 8). Similar results were also observed for BayesA (results not shown). These results indicate that the length of the genomic window used to model CS has minimal impact on the accuracy of the CS and LD-CS models. A possible reason is that only very few windows contain recombinations and most of the non-recombinant windows are not affected by window length. Fitting putative QTL at each SNP may show a significant advantage over e.g. 1-cM bins only when a larger number of observations is also available, to compensate for the much larger number of putative QTL allele effects to estimate.

## Discussion

The objectives of this study were (1) to develop a Bayesian statistical method to model CS information explicitly, (2) to study contributions of LD and CS information to accuracy of genomic prediction across unrelated families and validation generations without re-training, and (3) to study the effects of historical LD, recent $N_e$ and MAF of QTL on the advantage of modeling LD and CS explicitly in improving prediction accuracy. The major focus of this study was a theoretical exploration of contributions of LD and CS information to prediction accuracy in pedigree populations. The new CS and LD-CS model developed in this study enables precise disentanglement of CS information from LD since it models CS explicitly using allele origins. The simulation studies that were performed assist in this disentanglement by limiting genetic information to LD, CS and pedigree relationships, ruling out the noise that exists in real datasets (e.g. dominance, epistasis, imprinting, epigenetics). Investigation on real datasets is, however, worthy of future studies for potential applications of the CS and LD-CS models. In the following section, the mechanisms by which LD and CS information contribute to prediction accuracy across families and generations, as well as the effects of historical LD, recent $N_e$ and MAF of QTL on prediction accuracy are discussed.

### Explicit modeling of CS information

In this study, a new method that explicitly models CS information was developed for genomic prediction in pedigree populations. This method models transmission of putative QTL alleles within consecutive non-overlapping genomic windows of sufficiently small length (1 cM in this study), such that the recombination rate is so small that the alleles at all polymorphic loci within the window are expected to co-segregate for several generations. Co-segregation of QTL alleles was modeled using parental allele origins at SNPs that cover the genomic window, which are independent of the level of LD between QTL and SNPs. This method is applicable to any type of pedigree population provided that allele origins are available (or can be imputed) from founders to offspring. The method of modeling CS at putative QTL using allele origins at observable SNPs is similar to the method developed by Fernando and Grossman [29], but has the advantage that it allows estimation of values of founder alleles at putative QTL using single site Gibbs sampling. In Fernando and Grossman [29], the value of paternal and maternal alleles at putative QTL were fitted in the model for every pedigree individual to explain its breeding value. The value of QTL alleles is usually correlated among individuals that are related by the pedigree, and therefore, the estimation of these values was achieved by solving mixed model equations, which requires the inverse of the covariance matrix of these values for all pedigree members. Computation of the Fernando and Grossman [29] method is manageable for marker-assisted selection, where the number of molecular markers is usually small, but would not be feasible for genomic prediction. However, for genomic prediction using dense SNP panels, the CS model developed in this study is computationally tractable because the breeding values for all pedigree members are modeled using only the QTL alleles of pedigree founders, which are assumed independent and MCMC methods can be feasibly implemented to estimate their values.

The CS model (2) developed in this study can also be written as an equivalent breeding value model

7 $$\begin{aligned} \mathbf {y} = \mathbf {X}\varvec{{\upbeta }}+\mathbf {g}_{\text {CS}}+\mathbf {e}, \end{aligned}$$

where

$$\begin{aligned} \mathbf {g}_{\text {CS}} = \sum _{j=1}^{n_q}\mathbf {W}_j\mathbf {v}_j, \end{aligned}$$

and

$$\begin{aligned} \text {Var}(\mathbf {g}_{\text {CS}})&= \mathbf {G}_{\text {CS}} = \sum _{j=1}^{n_q}\mathbf {W}_j\mathbf {D}_j\mathbf {W}'_j,\quad \text { with}\\ \mathbf {D}_j&= \text {diag}\big \{\sigma ^2_{jk}\big \}_{k=1}^{n_j}. \end{aligned}$$

The covariance matrix of breeding values due to CS ($\mathbf {g}_{\text {CS}}$), $\mathbf {G}_{\text {CS}}$, quantifies the genetic covariance among individuals due to co-segregation at putative QTL. The genetic covariance between two individuals depends on the number of common founder alleles that the two individuals share through identity-by-descent, averaged across $n_q$ QTL, with the corresponding QTL effect variances as weights. This equivalent breeding value model (7) was used by Luan et al. [10] in their study on the contributions of CS and LD information to prediction accuracy in Italian Brown Swiss bulls. In Luan et al. [10], CS was modeled at each SNP locus of the Bovine SNP 50K chip, which were assumed to be surrogates of QTL. The covariance matrix $\mathbf {G}_{\text {CS}}$ was constructed independently at each SNP, and then averaged across all SNPs using equal weights. Compared to Luan et al. [10], the CS model developed herein has three advantages. First, the CS model (2) fits putative QTL within short genomic windows, which are much fewer than the number of SNPs in the 50K chip. Modeling CS at each SNP is not necessary because CS information is based on linkage and is conserved over longer genomic distances. Second, the CS model (2) allows different variances of QTL effects depending on their allele frequencies and sizes of the QTL effects. Larger QTL effects are estimated with less shrinkage, or equivalently larger weights in $\mathbf {G}_{\text {CS}}$. Third, the computation time for model (2) increases linearly with the number of individuals (*n*) times the number of founder QTL alleles $\big (\sum _{j=1}^{n_q}n_j\big )$, while for the mixed model approach in Luan et al. [10], computation time increases cubically with *n* because it requires the inverse of a dense matrix, $\mathbf {G}_{\text {CS}}$.

It is generally accepted that LD between QTL and SNPs, co-segregation of QTL with SNP alleles, and pedigree relationships at QTL captured by SNPs are the three main sources of information that contribute to the accuracy of genomic prediction [8–10, 16, 17, 34]. Most previous studies aimed at disentangling these three sources of information were based on multiple regression models on SNP genotypes (the LD model). The LD model only allows the part of CS information that is implicitly captured by SNP genotypes to be evaluated, which is highly variable depending on the number and density of SNPs, the level of historical LD, population structure and pedigree relationships. The CS model developed in this study enables precise disentanglement of CS from LD information because explicit modeling of CS information using parental allele origins does not depend on the level of LD between QTL and SNPs. As an intriguing consequence, results in this study are in contrast to some typical findings in several previous studies based on the LD model. For example, Habier et al. [9] showed that CS information captured by SNP genotypes contributed little to prediction accuracy across half-sib families and prediction accuracy decreased rapidly with increasing training size. In this study, prediction accuracy from the CS model persisted with increasing training size regardless of historical LD. This difference is mainly because Habier et al. [9] only considered the part of CS information that is implicitly captured by GBLUP, while the CS model in this study captures most of CS information due to modeling CS explicitly.

### Contributions of LD and CS information in half-sib designs

In the simulated datasets with different numbers of unrelated half-sib families, both LD and CS information contributed to the accuracy of genomic prediction. Accuracy of the LD model relies on the level of LD between QTL and SNPs in the base population. Accuracy of the CS model relies on accurate estimation of the value of founder QTL alleles that are transmitted to half-sib offspring within the same family. Accuracy of the LD model increased rapidly with increasing training size when LD between QTL and SNPs was high, because high LD is conserved across families and increasing training size brings in more data to improve estimation of SNP effects. However, when historical LD is low or zero, accuracy of the LD model mainly comes from capturing CS information, which only exists within the same half-sib family. Consequently, with more unrelated half-sib families included in the training population, accuracy of the LD model decreased and became lower than accuracy of the CS model. This is because only half-sibs from the same family contribute to prediction accuracy. In the CS model, the value of founder QTL alleles is estimated using only information from the same half-sib family, while the LD model estimates SNP effects by pooling CS information across all families, which is erroneous because linkage phase and LD is highly variable across a large number of unrelated families.

### Contributions of LD and CS information in extended pedigrees with different recent $N_e$

Three mating designs were simulated that differed in the number of parents per generation and the number of progeny per mating. Pedigrees 1 and 3 resemble the breeding program where a few sires are selected and intensively used for breeding in each generation. CS information made significant contributions to prediction accuracy in pedigrees 1 and 3, because a limited number of sire alleles segregated among a large number of their progeny, and the value of the sires’ alleles can be estimated accurately based on the amount of data available. Pedigree 1 is a balanced nested design with identical family sizes, which is similar to the structure of nucleus herds in swine [35] or poultry [5] breeding programs. Pedigree 3 is an unbalanced design with an influential sire in each generation that has more than 80 % of the total progeny, which resembles a dairy cattle population, where artificial insemination is widely used [36]. CS information had a larger contribution in pedigree 3 than in pedigree 1, because most progeny in a cohort inherited alleles from only one sire in pedigree 3. In contrast, pedigree 2 resembles an outbred population where all individuals survive and each mating has very few progeny. The number of unique parental alleles is large but each allele is transmitted only to very few progeny. As a result, the value of founder QTL alleles in pedigree 2 cannot be estimated accurately because each allele has only limited data available.

The difference between the three mating designs can be quantified by recent $N_e$. The $N_e$ of pedigrees 1 and 3 was less than 20, while that of pedigree 2 was close to 200. CS information has a larger contribution to prediction accuracy in a population with a smaller recent $N_e$ because individuals tend to be more closely related and share more founder alleles at QTL. The importance of CS information in the three mating designs was clearly illustrated in the scenario without historical LD among founders, where the long-term accuracy only stems from CS information. As shown in Figs. 5 and 6, the long-term accuracy by modeling CS explicitly was most persistent for pedigree 3, followed by pedigree 1, and least persistent for pedigree 2. A similar trend was also observed for the CS model when both LD and CS information contributed to prediction accuracy (Figs. 5 and 6).

The contribution of LD information to prediction accuracy should not depend on the mating design because high historical LD between QTL and SNPs is mostly between closely linked loci and hardly erodes within several recent generations. However, because the LD model also implicitly captures information from CS and pedigree relationships [9], accuracy of the LD model is affected by the mating design. For example, in the scenario with high historical LD, accuracy of the LD model was higher for pedigrees 1 and 3 than for pedigree 2 (Fig. 5). These results agree with Muir [19], who found that prediction accuracy of the GBLUP model decreased when recent $N_e$ increased, and this reduction was larger when QTL and SNPs were in linkage equilibrium (LE) than when they were in LD.

In general, when historical LD is high between QTL and SNPs, long-term accuracy is mostly contributed by LD information and CS information has little contribution regardless of recent $N_e$. However, when historical LD is low, CS information contributes most to long-term accuracy, especially when the mating design creates a very small recent $N_e$.

### The effect of MAF of QTL on contributions of LD and CS information

LD quantifies the correlation between allele states at QTL and SNPs. The LD model captures this correlation using multiple regression on SNP genotypes. Strong correlations can only exist when QTL and SNPs have similar MAF, as in the Common QTL scenario. Correlations are expected to be low when most QTL have low MAF, while SNPs have moderate MAF, as in the Rare QTL scenario. In the simulated datasets, the correlation between allele states exists in the form of historical LD and recent CS. Historical LD between closely linked loci is hardly eroded by recombination. The correlation generated by CS can exist between loci over long chromosome regions, which erodes fast with recombination. Both forms of correlation can be captured by the LD model, however, the LD model has persistent long-term accuracy only when historical LD between QTL and SNPs is high, which requires similar MAF between QTL and SNPs. When historical LD is low due to most QTL having low MAF, prediction accuracy of the LD model mainly comes from implicitly capturing CS information, which decreases rapidly across validation generations because CS information across long chromosome regions erodes fast with recombination. Similar results have been observed by Habier et al. [9] for the GBLUP model.

CS follows the transmission of QTL alleles among related individuals, which is independent of historical LD. As a result, prediction accuracy from the CS model is not affected by the level of historical LD. Accuracy due to CS information depends on (1) the length of the founder haplotypes that are used to follow transmission of putative QTL alleles, which determines the rate of erosion of CS due to recombination; and (2) the accuracy with which the value of founder alleles can be estimated, which depends on the amount of phenotype data for the progeny that inherited the same founder haplotype. The CS model that used haplotypes of length 1 cM is expected to have only a few recombinations within haplotypes, and therefore CS information contributes to long-term accuracy provided that the value of founder QTL alleles can be estimated correctly with sufficient data.

### The effect of prior distributions on prediction accuracy

Prior distributions in BayesA and BayesB [7] were used to allow simultaneous estimation of SNP effects ${\varvec{{\upalpha }}}$ in the LD model and the value of founder QTL alleles $\mathbf {v}_j$ in the CS model. GBLUP was also used in this study and had similar results as BayesA (results not shown). BayesA represents a method of shrinkage regression without variable selection. In BayesA, independent *t* prior distributions are given to $\alpha _l$ and $v_{jk}$. When using the posterior mode as the point estimate of a parameter $\beta$, the amount of shrinkage imposed by a scaled *t* prior distribution with degrees of freedom $\nu$ and scale parameter $S^2$, $t(0,\nu ,S^2)$, is proportional to $\log \Big (1+\frac{\beta ^2}{\nu S^2}\Big )$ [37]. The posterior mean used in this study is expected to be close to the mode due to the almost symmetric posterior distribution at convergence of the MCMC [31]. This means that the estimates of small $\beta$ are heavily shrunk towards zero but large $\beta$ are less shrunk. BayesB represents a variable selection method, in which the prior for each $\alpha _l$ and $v_{jk}$ is a mixture of a point mass at zero and a *t* distribution elsewhere. BayesB results in much heavier shrinkage towards zero than BayesA, and consequently the effective number of loci fitted in the model is larger in BayesA than in BayesB [9].

The effect of using alternate prior distributions on the LD model is twofold. When historical LD is high between QTL and SNPs, high prediction accuracy is usually achieved by the LD model, with nearly unbiased estimates of large SNP effects, while effectively shrinking small SNP effects towards zero. In the simulated datasets of this study, BayesB resulted in higher accuracy than BayesA because the number of QTL was much smaller than the number of SNPs. When historical LD is low, prediction accuracy mainly comes from implicitly capturing CS information, which depends on the effective number of SNPs fitted in the model. Then, BayesA results in higher accuracy than BayesB due to fitting relatively more SNPs, which can capture more CS information than BayesB [9].

The effect of prior distributions on the CS model does not depend on historical LD, but does depend on the MAF of QTL. When the MAF of QTL is high, BayesA tends to result in higher accuracy than BayesB because of fitting more founder alleles that co-segregate with common QTL alleles. When the MAF of QTL is low, BayesB tends to result in higher accuracy than BayesA, because only a small proportion of founder alleles carry QTL and their values can be estimated more accurately by variable selection of BayesB.

### Implementation of LD-CS model in field datasets

In real livestock populations, persistently high accuracy across validation generations using the LD model has rarely been observed [5, 16–18], which suggests that historical LD between QTL and SNPs is not perfect, and prediction accuracy relies more on CS than on LD information [10, 20]. The LD-CS model is recommended to improve long-term accuracy due to capturing both LD and CS information explicitly. Simulation results in this study suggest that the LD-CS model tends to result in the highest prediction accuracy of predictions in almost all scenarios. Using a similar LD-CS model as model (7), Luan et al. [10] show that in a pedigree population of Italian Brown Swiss bulls, LD information does not contribute to accuracy beyond that due to CS information. Recent studies in pig [28] and Atlantic salmon [38] breeding populations showed that the CS model of Luan et al. [10] had similar or lower accuracy than the GBLUP model, depending on trait heritability, SNP density, and the number of generations of pedigree data used to infer SNP allele origins. Results from field data suggest that in livestock populations, both CS and LD information contribute to prediction accuracy, and modeling CS explicitly can achieve almost the same accuracy as fitting SNP genotypes. Furthermore, the simulation results in this study suggest that modeling LD and CS explicitly improves prediction accuracy compared to modeling either LD or CS, when historical LD between QTL and SNPs is low due to most QTL being rare, as represented by the Rare QTL scenario.

There are several computational issues in implementing the CS or LD-CS models (2) in field datasets. First, obtaining parental allele origins from SNP genotypes for all pedigree members can be computationally prohibitive. This is usually achieved in two steps. SNP genotypes are first phased into haplotypes, which are then used to infer parental allele origins using pedigree information [12, 33]. Our results show that the advantage of modeling CS information explicitly in improving prediction accuracy depends strongly on the accuracy of allele origins, and errors in phasing and allele origin imputation can reduce or even nullify the advantage of the CS or LD-CD models. This problem can become less demanding with the availability of higher SNP density, genome re-sequencing, and identification of multi-allelic markers such as copy number variants and insertions/deletions. Second, the computation time for the MCMC algorithm of the CS model (2) increases with the number of pedigree founders, because the number of alleles at each putative QTL ($n_j$) is twice the number of founders. It is suggested that putative QTL be modeled at every 1 cM of the genome to reduce the total number of QTL alleles, as justified by the fact that recombination occurs very rarely within a 1-cM genomic window over several consecutive generations. Furthermore, instead of treating the values of QTL alleles as independent, they can be clustered according to their probability of identity-by-descent with respect to some historical common ancestors beyond the pedigree founders [33, 39, 40]. However, when the number of founders is large, the equivalent breeding value model (7) is recommended since the mixed model equations have the number of genotyped individuals as dimension.

## Conclusions

In this study, a new method that explicitly models co-segregation information was developed for genomic prediction of breeding values. Breeding values in this CS model were modeled as the sum of independent values of putative QTL alleles among pedigree founders, which were traced down in the pedigree using SNP haplotypes. When the training size was increased by adding unrelated half-sib families, accuracy of the CS model increased and plateaued, but accuracy of the LD model that fits SNP genotypes dropped when historical LD between QTL and SNPs was low. Modeling both LD and CS information improved prediction accuracy compared to modeling either LD or CS, especially when historical LD was low and recent CS information contributed substantially to prediction accuracy among families, which is probably the case for recent genomic evaluation in most livestock populations. The effects of recent $N_e$, historical LD, and MAF of QTL on persistence of accuracy across validation generations without retraining were investigated when explicitly modeling LD and CS information. The LD model had persistently high accuracy across validation generations only when historical LD between QTL and SNPs was high, which requires that QTL and SNPs have similar MAF. When historical LD between QTL and SNPs was low, accuracy of the LD model came mostly from capturing CS information, which was much lower and less persistent than that of the CS and LD-CS models. The contribution of CS information increased with smaller recent $N_e$, because the number of segregating QTL alleles of pedigree founders was smaller and their value could be estimated more accurately with sufficient data. Since the recent $N_e$ of most livestock populations is small and historical LD between QTL and SNPs tends to be low, modeling CS explicitly in addition to LD has potential to improve long-term accuracy provided that the allele origins can be accurately imputed.

## Additional files

10.1186/s12711-016-0255-4 Boundaries of linkage disequilibrium between a QTL and a SNP with different MAF. This file provides derivation and simulation results for boundaries of linkage disequilibrium between a QTL and a SNP with different MAF.

10.1186/s12711-016-0255-4 Bayesian inference for the LD-CS model. This file provides derivation of full conditional distributions of parameters in the LD-CD model, and MCMC algorithm to get point estimates of parameter values.
