# Supplementary material for: Contributions of linkage disequilibrium and co-segregation information to the accuracy of genomic prediction
Source: Genet Sel Evol. 2016 Oct 11;48:77. doi: 10.1186/s12711-016-0255-4 (PMC5060012; doi:10.1186/s12711-016-0255-4)
Supplement: Supplementary file 1 — 10.1186/s12711-016-0255-4 Boundaries of linkage disequilibrium between a QTL and a SNP with different MAF. This file provides derivation and simulation results for boundaries of linkage disequilibrium between a QTL and a SNP with different MAF. [file 12711_2016_255_MOESM1_ESM.pdf]

Additional file 1 — Boundaries of linkage disequilibrium between a QTL and a SNP with different MAF

The boundary of linkage disequilibrium (LD) between a bi-allelic SNP and a bi-allelic QTL is investigated when they have different minor allele frequencies (MAF).

Boundary of LD measured by D

Let M_1 and M_2 denote the two alleles of SNP with allele frequencies p_m and q_m, respectively, and Q_1 and Q_2 the two alleles at QTL with allele frequecies p_q and q_q, respectively. The D measure of LD is given by

$$\begin{aligned} D &= \mathrm{P}(M_1Q_1) - p_m p_q \\ &= -\mathrm{P}(M_1Q_2) + p_m q_q \\ &= -\mathrm{P}(M_2Q_1) + q_m p_q \\ &= \mathrm{P}(M_2Q_2) - q_m q_q, \end{aligned}$$

where $\mathrm{P}(M_iQ_j)$ is the frequency of haplotype M_iQ_j, $i, j \in \{1, 2\}$. It follows that

$$\begin{aligned} \mathrm{P}(M_1Q_1) &= D + p_m p_q, \\ \mathrm{P}(M_1Q_2) &= p_m q_q - D, \\ \mathrm{P}(M_2Q_1) &= q_m p_q - D, \\ \mathrm{P}(M_2Q_2) &= D + q_m q_q. \end{aligned}$$

Since $\mathrm{P}(M_1Q_1)$, $\mathrm{P}(M_1Q_2)$, $\mathrm{P}(M_2Q_1)$, and $\mathrm{P}(M_2Q_2)$ are non-negative, D should satisfy all the following inequalities

$$\begin{aligned} D + p_m p_q &\geq 0, \\ p_m q_q - D &\geq 0, \\ q_m p_q - D &\geq 0, \\ D + q_m q_q &\geq 0. \end{aligned}$$

Therefore

$$-\min\{p_m p_q, q_m q_q\} \leq D \leq \min\{p_m q_q, q_m p_q\}.$$

When M_2 and Q_2 are minor alleles, i.e. $q_m \leq p_m$ and $q_q \leq p_q$, we have

$$-q_m q_q \leq D \leq \min\{p_m q_q, q_m p_q\}, \tag{1}$$

because of $q_m q_q \leq p_m p_q$.

Boundary of LD measured by r

LD can also be measured as the correlation coefficient r between genotypes of the SNP and QTL. The quantity r is preferred over D because the range of the value D takes is highly variable depending on p_m and p_q, but the value of r always ranges between -1 and 1. Furthermore, the value r^2 quantifies the proportion of QTL genetic variance that can be explained by SNP genotypes, which is more relevant to the interpretation of SNP regression model.

It can be shown that

$$r = \frac{D}{\sqrt{p_m q_m p_q q_q}}.$$

Following equation (1), the range of r between SNP and QTL is given by

$$-\sqrt{\frac{q_m q_q}{p_m p_q}} \leq r \leq \min\left\{\sqrt{\frac{p_m q_q}{q_m p_q}}, \sqrt{\frac{q_m p_q}{p_m q_q}}\right\}.$$

The ranges of r at different MAF of QTL are illustrated in Figure 1 when the MAF of SNP equals 0.5, 0.25, 0.05 and 0.01, respectively.

When the MAF of QTL decreases to zero, the range of r also shrinks to zero, indicating that high LD is impossible when the MAF of either QTL or SNP is close to zero. Further, perfect LD ($r = 1$) is only attainable when QTL and SNP have the same MAF, as shown by dashed vertical lines in Figure 1.

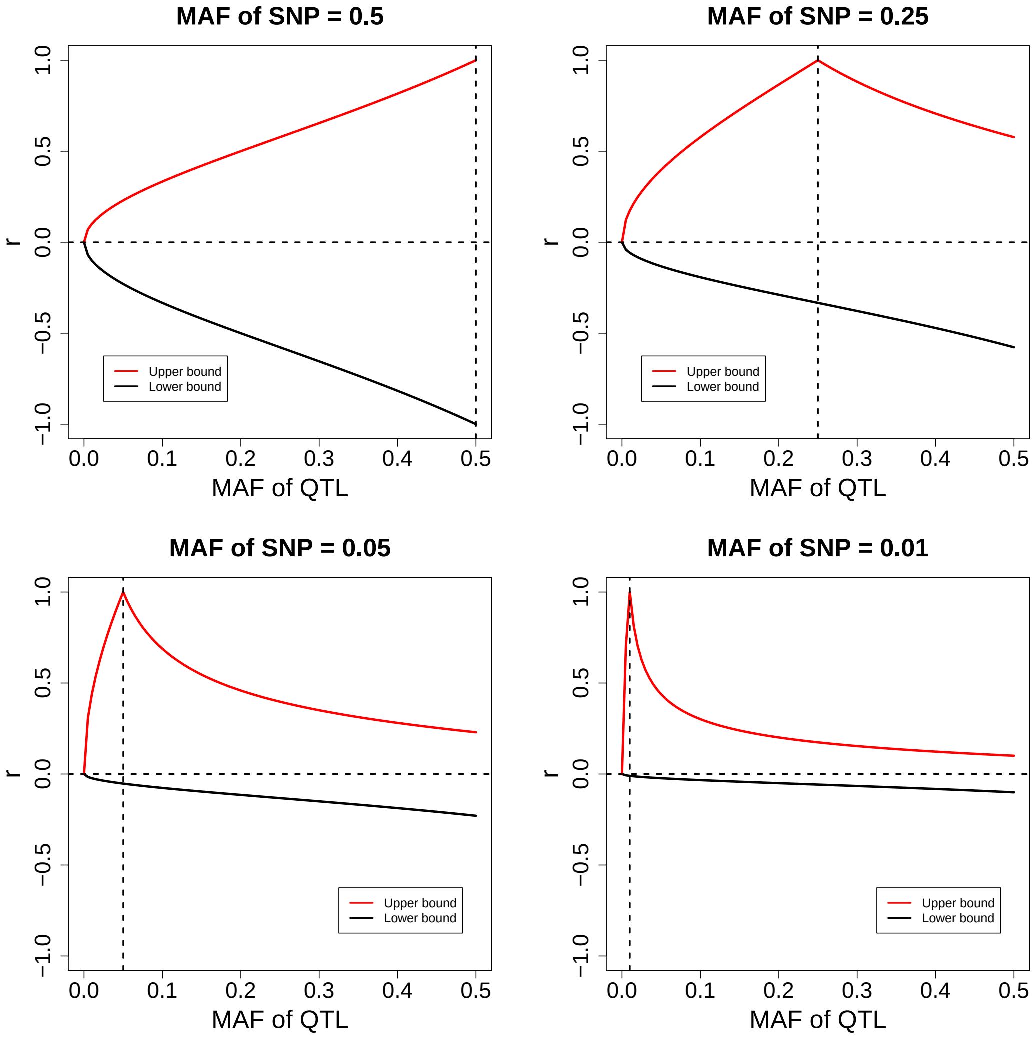

Figure 1: **Boundaries of r measure of LD between a QTL and a SNP when the MAF of SNP equals 0.5, 0.25, 0.05 and 0.01.**
