# Supplementary material for: Contributions of linkage disequilibrium and co-segregation information to the accuracy of genomic prediction
Source: Genet Sel Evol. 2016 Oct 11;48:77. doi: 10.1186/s12711-016-0255-4 (PMC5060012; doi:10.1186/s12711-016-0255-4)
Supplement: Supplementary file 2 — 10.1186/s12711-016-0255-4 Bayesian inference for the LD-CS model. This file provides derivation of full conditional distributions of parameters in the LD-CD model, and MCMC algorithm to get point estimates of parameter values. [file 12711_2016_255_MOESM2_ESM.pdf]

# Additional file 2 — Bayesian inference for the LD-CS model

This document gives a description of how the Bayesian methods "BayesA" and "BayesB" in Meuwissen et al. (2001) were adapted for inference using the LD-CS model. Inference using either the LD or CS model is straightforward by excluding the CS or LD term from the LD-CS model, respectively.

In this study, $\mathbf{X} = \mathbf{1}$ is a vector of ones with length $n$, and $\boldsymbol{\beta} = \mu$ is the overall mean for all training individuals. Indicator variables $\delta_l$ for $\alpha_l$ $(l = 1, 2, \cdots, m)$ and $\psi_{jk}$ for $v_{jk}$ $(k = 1, 2, \cdots, n_j)$ are introduced, to indicate when the corresponding random effect is fitted in the model; $\delta_l$ or $\psi_{jk}$ equals 1 when the effect is included in the model and 0 otherwise.

The residual sampling distribution for $\boldsymbol{y}$ is multivariate normal:

$$\boldsymbol{y}|\mu, \boldsymbol{\alpha}, \boldsymbol{v}_j, \sigma_e^2 \sim \mathrm{N}\left(\mathbf{1}\mu + \mathbf{Z}\boldsymbol{\Delta}\boldsymbol{\alpha} + \sum_{j=1}^{n_q} \mathbf{W}_j \boldsymbol{\Psi}_j \boldsymbol{v}_j, \mathbf{I}\sigma_e^2\right), \tag{1}$$

where

$$\begin{aligned} \boldsymbol{\Delta} &= \mathrm{Diagonal}\{\delta_l\}_{l=1}^{m}, \\ \boldsymbol{\Psi}_j &= \mathrm{Diagonal}\{\psi_{jk}\}_{k=1}^{n_j}, \end{aligned}$$

where $n_j$ is the dimension of vector $\boldsymbol{v}_j$.

The prior distribution for $\mu$ is improper uniform, $\pi(\mu) \propto$ constant. The prior distribution for $\sigma_e^2$ is a scaled inverse chi-square distribution with degrees of freedom $\nu_e$ and scale parameter $S_e^2$:

$$\pi(\sigma_l^2) = \chi_{\nu_e}^{-2}(S_e^2),$$

where $\chi_a^{-2}(b)$ is probability density function of scaled inverse chi-square distribution with $a$ degrees of freedom and scale parameter $b$.

The prior distribution for $\alpha_l$ conditional on its variance $\sigma_l^2$ is normal:

$$\pi(\alpha_l|\sigma_l^2) = \mathrm{N}(0, \sigma_l^2),$$

and the prior distribution of $\sigma_l^2$ is a scaled inverse chi-square distribution:

$$\pi(\sigma_l^2) = \chi_{\nu_\alpha}^{-2}(S_\alpha^2), \quad l = 1, 2, \cdots, m.$$

The prior distribution for $v_{jk}$ conditional on its variance $\sigma_{jk}^2$ is normal:

$$\pi(v_{jk}|\sigma_{jk}^2) = \mathrm{N}(0, \sigma_{jk}^2),$$

and the prior distribution of $\sigma_{jk}^2$ is a scaled inverse chi-square distribution:

$$\pi(\sigma_{jk}^2) = \chi_{\nu_c}^{-2}(S_c^2), \quad j = 1, 2, \cdots, n_q, \text{ and } k = 1, 2, \cdots, n_j.$$

The prior distributions for $\delta_l$ and $\psi_{jk}$ are Bernoulli distributions:

$$\begin{aligned} \pi(\delta_l) &= \left(1 - \pi_{\mathrm{SNP}}\right)^{\delta_l} \pi_{\mathrm{SNP}}^{(1-\delta_l)}, \quad l = 1, 2, \cdots, m, \\ \pi(\psi_{jk}) &= \left(1 - \pi_{\mathrm{CSE}}\right)^{\psi_{jk}} \pi_{\mathrm{CSE}}^{(1-\psi_{jk})}, \quad j = 1, 2, \cdots, n_q, \text{ and } k = 1, 2, \cdots, n_j, \end{aligned}$$

where $\pi_{\mathrm{SNP}}$ and $\pi_{\mathrm{CSE}}$ are the expected proportions of SNPs and founder QTL alleles that have null effects on the trait, respectively. Method BayesB assumes that $\pi_{\mathrm{SNP}} \in (0, 1)$ and $\pi_{\mathrm{CSE}} \in (0, 1)$, while method BayesA assumes that $\pi_{\mathrm{SNP}} = 0$ and $\pi_{\mathrm{CSE}} = 0$.

The joint posterior distribution for model parameters $\boldsymbol{\theta} = \big(\mu, \boldsymbol{\alpha}, \boldsymbol{v}_{jk}, \boldsymbol{\sigma}_l^2, \boldsymbol{\sigma}_{jk}^2, \sigma_e^2, \boldsymbol{\delta}_l, \boldsymbol{\psi}_{jk}\big)'$ is given by

$$
\begin{aligned}
p(\boldsymbol{\theta}|\boldsymbol{y}) \propto & L\big(\boldsymbol{y}|\mu, \boldsymbol{\alpha}, \boldsymbol{v}_{jk}, \boldsymbol{\delta}_l, \boldsymbol{\psi}_{jk}, \sigma_e^2\big)\pi\big(\boldsymbol{\alpha}|\boldsymbol{\sigma}_l^2\big)\pi\big(\boldsymbol{\sigma}_l^2\big)\pi\big(\boldsymbol{v}_{jk}|\boldsymbol{\sigma}_{jk}^2\big)\pi\big(\boldsymbol{\sigma}_{jk}^2\big)\pi\big(\boldsymbol{\delta}_l\big)\pi\big(\boldsymbol{\psi}_{jk}\big)\pi\big(\sigma_e^2\big) \\
\propto & \big(\sigma_e^2\big)^{-n/2} \exp\left\{ -\frac{1}{2\sigma_e^2} \sum_{i=1}^{n} \left( y_i - \mu - \sum_{l=1}^{m} \boldsymbol{z}_l \delta_l \alpha_l - \sum_{j=1}^{n_q} \sum_{k=1}^{n_j} \boldsymbol{w}_{jk} \psi_{jk} v_{jk} \right)^2 \right\} \\
& \times \prod_{l=1}^{m} \big(\sigma_l^2\big)^{-1/2} \exp\left\{ -\frac{\alpha_l^2}{2\sigma_l^2} \right\} \times \prod_{l=1}^{m} \big(\sigma_l^2\big)^{1+\nu_\alpha/2} \exp\left\{ -\frac{\nu_\alpha S_\alpha^2}{2\sigma_l^2} \right\} \\
& \times \prod_{j=1}^{n_q} \prod_{k=1}^{n_j} \big(\sigma_{jk}^2\big)^{-1/2} \exp\left\{ -\frac{v_{jk}^2}{2\sigma_{jk}^2} \right\} \times \prod_{j=1}^{n_q} \prod_{k=1}^{n_j} \big(\sigma_{jk}^2\big)^{1+\nu_c/2} \exp\left\{ -\frac{\nu_c S_c^2}{2\sigma_{jk}^2} \right\} \\
& \times \prod_{l=1}^{m} \pi_{\text{SNP}}^{(1-\delta_l)} \big(1 - \pi_{\text{SNP}}\big)^{\delta_l} \times \prod_{j=1}^{n_q} \prod_{k=1}^{n_j} \pi_{\text{SNP}}^{(1-\psi_{jk})} \big(1 - \pi_{\text{CSE}}\big)^{\psi_{jk}} \\
& \times \big(\sigma_e^2\big)^{1+\nu_e/2} \exp\left\{ -\frac{\nu_e S_e^2}{2\sigma_e^2} \right\},
\end{aligned}
\tag{2}
$$

where $L\big(\boldsymbol{y}|\mu, \boldsymbol{\alpha}, \boldsymbol{v}_{jk}, \boldsymbol{\delta}_l, \boldsymbol{\psi}_{jk}, \sigma_e^2\big)$ is the probability density function of the multivariate normal distribution of (1).

Inference on each of the model parameters $\boldsymbol{\theta}$ is based on random samples from its marginal posterior distribution, which are obtained through Gibbs sampling from the full conditional posterior distributions of each element in $\boldsymbol{\theta}$. The full conditional distribution for each parameter in $\boldsymbol{\theta}$.

The full conditional distribution for $\mu$ is normal:

$$
\pi(\mu|\cdot, \boldsymbol{y}) = \text{ N}\left( \frac{1}{n}\boldsymbol{1}'\boldsymbol{y}_\mu^*, \frac{\sigma_e^2}{n} \right),
$$

where

$$
\boldsymbol{y}_\mu^* = \boldsymbol{y} - \sum_{l=1}^{m} \boldsymbol{z}_l \delta_l \alpha_l - \sum_{j=1}^{n_q} \sum_{k=1}^{n_j} \boldsymbol{w}_{jk} \psi_{jk} v_{jk},
$$

and $\cdot$ denotes all the other parameters in $\boldsymbol{\theta}$ except for $\mu$. Similar rule applies in the following.

The full conditional distribution for $\alpha_l$ is normal:

$$
\pi(\alpha_l|\cdot, \boldsymbol{y}) = \text{ N}\left( \frac{\boldsymbol{z}_l'\boldsymbol{y}_l^*}{\boldsymbol{z}_l'\boldsymbol{z}_l + \lambda_l}, \frac{\sigma_e^2}{\boldsymbol{z}_l'\boldsymbol{z}_l + \lambda_l} \right),
$$

where

$$
\boldsymbol{y}_l^* = \boldsymbol{y} - \sum_{l' \neq l}^{m} \boldsymbol{z}_{l'} \delta_{l'} \alpha_{l'} - \sum_{j=1}^{n_q} \sum_{k=1}^{n_j} \boldsymbol{w}_{jk} \psi_{jk} v_{jk},
$$

and

$$
\lambda_l = \frac{\sigma_e^2}{\sigma_l^2}.
$$

The full conditional distribution for $v_{jk}$ is normal distribution

$$
\pi(v_{jk}|\cdot, \boldsymbol{y}) = \text{ N}\left( \frac{\boldsymbol{w}_{jk}'\boldsymbol{y}_{jk}^*}{\boldsymbol{w}_{jk}'\boldsymbol{w}_{jk} + \tau_{jk}}, \frac{\sigma_e^2}{\boldsymbol{w}_{jk}'\boldsymbol{w}_{jk} + \tau_{jk}} \right),
$$

where

$$\boldsymbol{y}^*_{jk} = \boldsymbol{y} - \sum_{l=1}^{m} \boldsymbol{z}_l \delta_l \alpha_l - \sum_{j' \neq j}^{n_q} \sum_{k' \neq k}^{n_{j'}} \boldsymbol{w}_{j'k'} \psi_{j'l'} v_{j'k'},$$

and

$$\tau_{jk} = \frac{\sigma_e^2}{\sigma_{jk}^2}.$$

The full conditional distribution for $\sigma_l^2$ is scaled inverse chi-square distribution

$$\pi(\sigma_l^2 | \cdot, \boldsymbol{y}) = \chi^{-2}_{\tilde{\nu}_\alpha}\big(\tilde{S}^2_\alpha\big),$$

where

$$\tilde{\nu}_\alpha = \nu_\alpha + 1,$$
$$\tilde{S}^2_\alpha = \frac{\nu_\alpha S^2_\alpha + \alpha_l^2}{\nu_\alpha + 1}.$$

The full conditional distribution for $\sigma_{jk}^2$ is scaled inverse chi-square distribution

$$\pi(\sigma_{jk}^2 | \cdot, \boldsymbol{y}) = \chi^{-2}_{\tilde{\nu}_c}\big(\tilde{S}^2_c\big),$$

where

$$\tilde{\nu}_c = \nu_c + 1,$$
$$\tilde{S}^2_c = \frac{\nu_c S^2_c + v_{jk}^2}{\nu_c + 1}.$$

The full conditional distribution for $\sigma_e^2$ is scaled inverse chi-square distribution

$$\pi(\sigma_e^2 | \cdot, \boldsymbol{y}) = \chi^{-2}_{\tilde{\nu}_e}\big(\tilde{S}^2_e\big),$$

where

$$\tilde{\nu}_e = \nu_e + n,$$
$$\tilde{S}^2_e = \frac{(\boldsymbol{y}^*)'(\boldsymbol{y}^*)}{\nu_e + n},$$

where

$$\boldsymbol{y}^* = \boldsymbol{y} - \mathbf{1}\mu - \sum_{l=1}^{m} \boldsymbol{z}_l \delta_l \alpha_l - \sum_{j=1}^{n_q} \sum_{k=1}^{n_j} \boldsymbol{w}_{jk} \psi_{jk} v_{jk}.$$

The full conditional distribution for $\delta_l$ is Bernoulli distribution with success probability

$$\Pr(\delta_l = 1 | \cdot, \boldsymbol{y}) = \frac{1}{1 + \exp\big(\log[h_0] - \log[h_1]\big)},$$

where

$$\log[h_1] = \big(1 - \pi_{\text{SNP}}\big) \exp\bigg\{ -\frac{1}{2\sigma_e^2} \big(\boldsymbol{y}^*_l - \boldsymbol{z}_l \alpha_l\big)' \big(\boldsymbol{y}^*_l - \boldsymbol{z}_l \alpha_l\big) \bigg\},$$
$$\log[h_0] = \pi_{\text{SNP}} \exp\bigg\{ -\frac{1}{2\sigma_e^2} \boldsymbol{y}^{*\prime}_l \boldsymbol{y}^*_l \bigg\},$$

with $\boldsymbol{y}_l^*$ as previously defined.

The full conditional distribution for $\psi_{jk}$ is Bernoulli distribution with success probability

$$\Pr(\psi_{jk} = 1|\cdot, \boldsymbol{y}) = \frac{1}{1 + \exp\big(\log[g_0] - \log[g_1]\big)},$$

where

$$\log[g_1] = \big(1 - \pi_{\text{CSE}}\big) \exp\Bigg\{ -\frac{1}{2\sigma_e^2}\big(\boldsymbol{y}_{jk}^* - \boldsymbol{w}_{jk}v_{jk}\big)'\big(\boldsymbol{y}_{jk}^* - \boldsymbol{w}_{jk}v_{jk}\big)\Bigg\},$$
$$\log[g_0] = \pi_{\text{CSE}} \exp\Bigg\{ -\frac{1}{2\sigma_e^2}\boldsymbol{y}_{jk}^{*\,\prime}\boldsymbol{y}_{jk}^*\Bigg\},$$

with $\boldsymbol{y}_{jk}^*$ as previously defined.

Values for hyperparameters $\nu_\alpha$, $\nu_c$ and $\nu_e$ were 4.2. For the LD and CS models, values of $S_\alpha^2$ and $S_c^2$ were chosen such that

$$\frac{\nu_\alpha S_\alpha^2}{\nu_\alpha - 2} = \frac{h^2 V_P}{2(1 - \pi_{\text{SNP}})\sum_{l=1}^m p_l(1-p_l)}, \qquad \text{and}$$
$$\frac{\nu_c S_c^2}{\nu_c - 2} = \frac{h^2 V_P}{(1 - \pi_{\text{CSE}})n_q},$$

respectively. For the LD-CS model, values of $S_\alpha^2$ and $S_c^2$ were chosen such that

$$\frac{\nu_\alpha S_\alpha^2}{\nu_\alpha - 2} = \frac{0.5h^2 V_P}{2(1 - \pi_{\text{SNP}})\sum_{l=1}^m p_l(1-p_l)}, \qquad \text{and}$$
$$\frac{\nu_c S_c^2}{\nu_c - 2} = \frac{0.5h^2 V_P}{(1 - \pi_{\text{CSE}})n_q},$$

where $V_P$ is the phenotypic variance in the training population, $p_l$ is the MAF of SNP $l$ in the training population, and $h^2$ is the trait heritability that equaled 0.3 in the simulations. The value of $S_e^2$ was chosen such that

$$\frac{\nu_e S_e^2}{\nu_e - 2} = (1 - h^2)V_P.$$
